# Lithobius (Chinobius) yuchernovi, a new lithobiid species from northeastern Siberia and the Kamchatka Peninsula, Russia (Chilopoda, Lithobiomorpha)

**DOI:** 10.3897/zookeys.693.14769

**Published:** 2017-08-23

**Authors:** Gyulli Sh. Farzalieva, Pavel S. Nefediev, Ivan H. Tuf

**Affiliations:** 1 Department of Invertebrate Zoology and Aquatic Ecology, Perm State University, Bukireva 15, Perm 614600, Russia; 2 Department of Ecology, Biochemistry and Biotechnology, Altai State University, Lenina 61, Barnaul 656049, Russia; 3 Department of Ecology and Environmental Sciences, Faculty of Science, Palacký University, Šlechtitelů 27, Olomouc 77900, Czech Republic; 4 Biological Institute, Tomsk State University, Lenina 36, Tomsk 634050, Russia

**Keywords:** *Lithobius*, new species, nomina nova, Russian Far East, Siberia, taxonomy

## Abstract

*Lithobius yuchernovi* **sp. n.** is described, based on type material from the Ola Plateau, Magadan Region, Russia. The new species is widely distributed in northeastern Siberia, ranging from the Magadan Region, until the eastern Chukot Autonomous Region and the Kamchatka Peninsula in the southeast, whence non-type material is documented. This species belongs to the subgenus Chinobius Matic, 1973 based on the structure of the female gonopodal claw (more than two denticles on the internal ridge). It differs from all Palearctic species of the genus *Lithobius* by the following apomorphy: distoventral tubercle supporting a cluster of long and curved setae situated on the last male tibia. In addition, it shows dorsal and ventral sulci on the last legs. New replacement names are introduced: *Lithobius zachiui* **nom. n.** for Lithobius (Chinobius) orientalis (Matic, 1973) and *Lithobius carli* **nom. n.** for Lithobius (Alokobius) orientalis Attems, 1953.

## Introduction

Northeastern Siberia, Russia, is a large territory lying east of the Lena and Aldan rivers and extending until the Bering Strait and the shores of the Bering Sea, between the Arctic and Pacific oceans. The Kamchatka Peninsula borders northeastern Siberia from the south and belongs to the Kamchatka-Kurilian geographical province (Davydova et al. 1960).

The lithobiomorph fauna of both regions is still poorly studied. To date, northeastern Siberia and the Kamchatka Peninsula are known to support only four species of one genus (Zalesskaja 1978, Eason 1996). A new species of lithobiid centipede currently has been recorded from the Ola Plateau highlands, Magadan Region. Additional material from that region, as well as the Chukot Autonomous Region and the Kamchatka Peninsula, mostly deposited in the Zoological Museum of the Lomonosov Moscow State University, Russia, shows this new species to actually be widespread in the area.

The present paper provides a description of the new species, with short comments on the taxonomic problems encountered in the subgenus Chinobius Matic, 1973 it belongs to. Additionally, new replacement names are introduced for junior homonyms.

## Materials and methods

Fifty-four specimens of both sexes of the new species treated below were collected from the Ola Plateau highlands, Magadan Region, by O.L. Makarova and A.B. Babenko. The type locality of the new species (Ola Plateau) is situated 130 km inland from the Sea of Okhotsk coast, northwest of the city of Magadan. It occupies an area of about 100 km^2^ (Yurtsev and Khokhryakov 1975) and consists of flat-topped mountains (photographs and detailed descriptions of typical habitats see in Makarova et al. 2014) mainly formed by Tertiary basalts, with a well-developed river network. The altitudes of the plateau average 1100–1600 m (hereafter, all altitudes are given above sea-level), with some peaks reaching 2000 m. The tundra belt in the study area (the upper reaches of the Ola River, 60°39'N, 151°16'E) begins from 900–1200 m, depending on slope exposition.

Additional non-type material (139 specimens) was collected from several localities in the Magadan Region, the Chukot Autonomous Region, and the Kamchatka Peninsula.

Material is currently deposited in the collections of the Zoological Museum of the Lomonosov Moscow State University, Moscow, Russia (ZMUM), the Perm State University, Perm, Russia (PSU), and the Manchester Museum of the University of Manchester, UK (MMUM).

The terminology of the external anatomy follows Bonato et al. (2010).

Measurements. The total body length was measured from the fore margin of the cephalic plate to the posterior end of the postpedal tergite. Leg length was measured excluding the length of the claw. Lengths are given as the minimum and maximum values. All measurements are given in millimeters (mm).

Plectrotaxy. Legs spinulation data are given in a separate table for holotype only. The number of coxal pores on legs 12–15 is presented in a formula where a sequence of Arabic numerals means the number of pores on these legs, respectively.

SEM micrographs were prepared at the PSU using a Hitachi TM3000 scanning electron microscope with a back-scatter electron detector. The drawings were executed by G. Sh. Farzalieva using a Meiji EMZ-5 stereo microscope and a RA-5 drawing tube.

### The following abbreviations are used in the text and table:

**V** ventral

**D** dorsal

**T**, **TT** tergite, tergites

**S** sternite

**C** coxa

**t** trochanter

**P** prefemur

**F** femur

**Ti** tibia

**Ts1** tarsus 1

**Ts2** tarsus 2

**a** anterior

**m** median

**p** posterior

## Results

### Taxonomy

#### Lithobius (Chinobius) yuchernovi sp. n.

Taxon classificationAnimaliaLithobiomorphaLithobiidae

http://zoobank.org/70178FA1-C117-4FFA-B915-C47783BA339B

Figs 1–9 , 10–12 , 13–20 , 21–28

##### Type material.

Holotype ♂ (ZMUM): Russia, Magadan Region, Kolyma Uplands, Ola Plateau highlands, 60°39'N, 151°16'E, nival community with *Cassiope tetragona*, 1275 m, 9.08.2011, leg. O.L. Makarova and A.B. Babenko.

**Paratypes**: 3 ♂♂, 4 ♀♀ (ZMUM), 2 ♂♂, 1♀ (MMUM, No. G7593), same data as holotype; 5 ♂♂, 4 ♀♀ (PSU), same locality, grass meadow, forb meadow with abundant legumes, nival community with *Cassiope tetragona*, dryas-forb tundra and ridge-top dryas-moss tundra, ca 1225–1470 m, 8–9.08.2011, leg. O.L. Makarova and A.B. Babenko; 5 ♂♂, 5 ♀♀ (ZMUM), 3 ♂♂, 12 ♀♀ (PSU), same locality, along bed of a nameless stream, at timber line, snowbed at ca 1150 m, 10.08.2011, leg. O.L. Makarova and A.B. Babenko; 3 ♂♂, 6 ♀♀ (PSU), same locality, forest belt of Ola Valley, willow bog, ca 820 m, 10.08.2011, leg. O.L. Makarova.

**Non-type material**: Chukot Autonomous Region: 6 ♂♂, 5 ♀♀, 1 subadult ♀ (ZMUM), environs of Anadyr Town, Maria Hill, under stones, 14–17.07.1971, leg. A.L. Tikhomirova and V.A. Turchaninova; 1 ♂, 7 ♀♀ (ZMUM), upper reaches of Bolshaya Osinovaya River, 66°52'N, 175°13'E, 30.07.1992, leg. D.I. Berman; 1 ♂ (ZMUM), near Markovo, 64°41'N, 170°26'E, spring alluvium of Anadyr River, 9.07.1971, leg. A.L. Tikhomirova; 8 ♂♂, 1 subadult ♂, 1 ♀♀ (ZMUM), Anadyr River, estuary of Balaganchik River, 64°54'N, 168°36'E, no date, leg. P.S. Tomkovich; 3 ♂♂, 16 ♀♀, 11 epimorph juveniles (PSU), Chaun Bay, Mt. Neitlin, 69°19'N, 171°27'E, pitfall traps, 5–25.08.1992, leg. D.I. Berman. Kamchatka Peninsula: 1 ♂ (ZMUM), near Elizovo, 53°10'N, 158°28'E, *Betula* forest with Poaceae, 11.08.1987, leg. A.V. Tanasevitch; 1 ♂, 1 ♀ (ZMUM), Kronotsky Nature Reserve, near Valley of Geysers, 54°31'N, 159°48'E, mountain tundra with mosses and lichens,1200 m, 2–3.09.1978, leg. A.V. Tanasevitch; 1 ♂, 3 ♀♀ (ZMUM), same locality, multiherbaceous *Betula* forest on slope, 1.09.1978, leg. A.V. Tanasevitch; 2 ♀♀ (ZMUM), southern border of Kronotsky Nature Reserve, Zhupanovo, *Abies* forest, 30.08.1987, leg. A.V. Tanasevitch. Magadan Region: 19 ♂♂, 49 ♀♀, Tenkinsky District, near Sibik-Tyellakh, “Aborigen” Field Station, 61°54'N, 149°18'E, Mt. Medvezhya, no date, leg. D.I. Berman; 1 ♂, 1 ♀ (ZMUM), Annachag Mts, Jack London Lake, 62°04'N, 149°31'E, ca 800 m, litter, 5–6.08.1985, leg. Lyubimova.

##### Name.

The new species honours Academician **Yu**ry Ivanovich **Chernov** (1934–2012), the outstanding researcher of the Arctic (Reznikova 2012).

##### Diagnosis.

A species of the genus Lithobius Leach, 1814, subgenus Chinobius Matic, 1973, normally with 20+20 elongate antennal articles; 9–11 ocelli, arranged in three rows; Tömösváry’s organ similar in size to the nearest ocellus; 2+2 teeth and setiform porodonts at dental margin of coxosternite; tergites without processes at posterior angles; tarsi 2-segmented, articulation being well-defined on all legs; legs 14–15 with DCa, leg 15 with an accessory apical claw; female gonopods with 2+2 spurs, gonopodal claw with 2–3 poorly-expressed denticles on internal ridge and with a single well-defined denticle on external ridge; last pair of legs swollen in male, ventrodistally with a group of curved setae on a round tubercle on tibiae, as well as shallow dorsal and ventral sulci on femora and tibiae.

##### Distribution

**(Figure 29).** Northeastern Siberia and the Kamchatka Peninsula.

##### Description.

**Figures 1–9.**
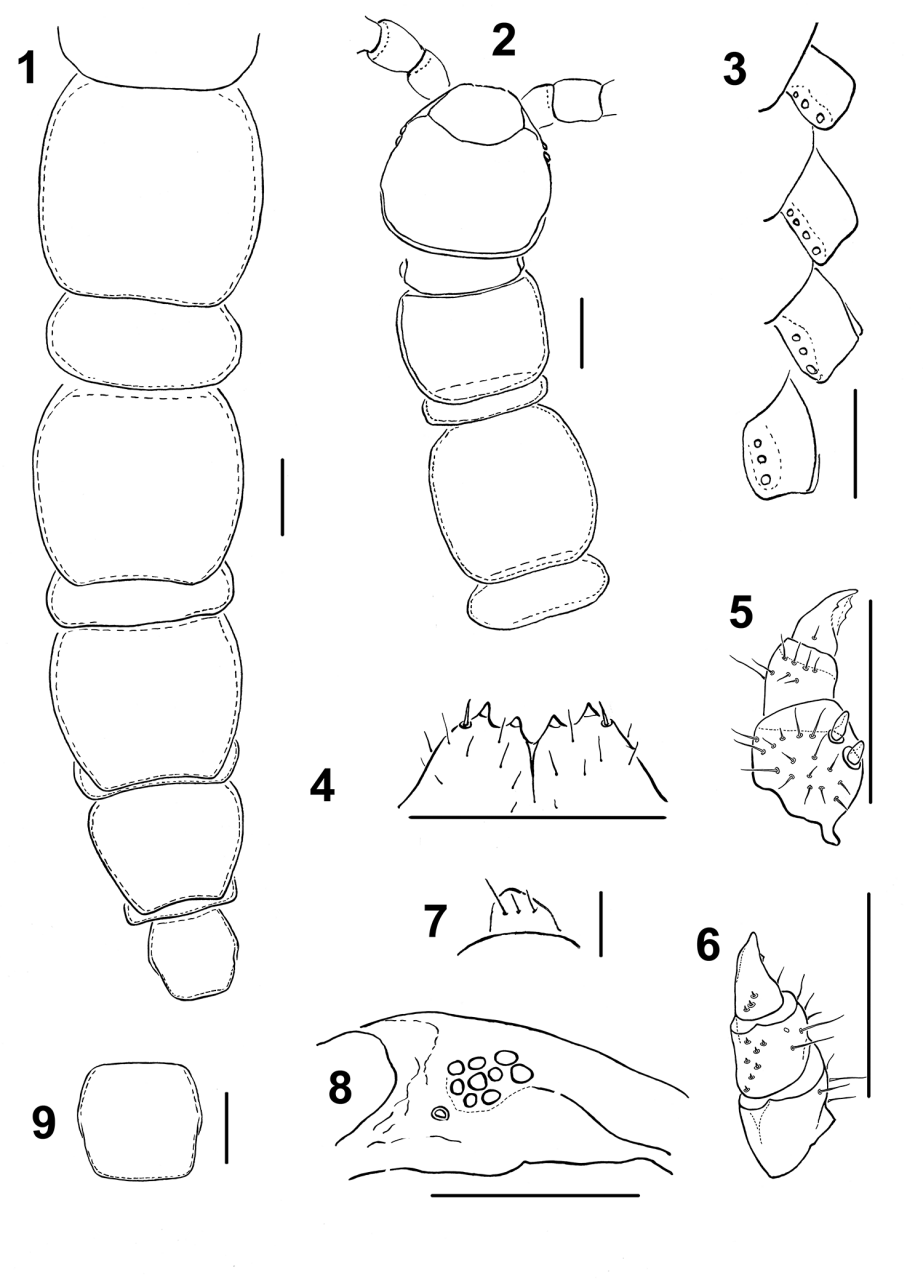
Lithobius (Chinobius) yuchernovi sp. n., paratypes. **1** male 8–16 tergites, dorsal view **2** male front body part, dorsal view **3** female coxal pores of legs 12–15, ventral view **4** male forcipular coxosternite, ventral view **5–6** female gonopod, ventral and dorsal view, respectively **7** male gonopod, ventral view **8** female ocelli and Tömösváry’s organ, lateral view **9** female intermediate tergite, dorsal view. Scale bars: 0.5 mm (**1–6, 8, 9**), 0.1 mm (**7**).

Holotype ♂. Body 12.9 mm long; colour in alcohol yellow-brownish, with a distinct, darker, axial stripe on forcipular T–T 10, thereafter axial stripe poorly-expressed. Tergites: almost smooth, without visible setae, T 15 distinct; posterior angles rounded from forcipular T to T 9; posterior margin of TT 10, 12 and 14 slightly sinuate; TT 9, 11, 13 and 15 without triangular projections, but TT 13 and 15 with posterior angles slightly drawn back (Fig. 1); posterior margin of intermediate T straight, breadth/length ratio 1.04 (length 0.68 mm, breadth 0.70 mm); T 10 broadened, breadth/length ratio 1.16 (length 1.23 mm, breadth 1.43 mm). Sternites: sparsely setose, breadth/length ratio of S 10, 1.23 (length 0.88 mm, breadth 1.08 mm); breadth/length ratio of S 15, 0.65 (length 0.55 mm; genital sternite more densely setose, as in Fig. 18).

Cephalic plate: breadth/length ratio 1.04 (length 1.20 mm, breadth 1.23 mm); much broader than forcipular T (breadth 0.90 mm) (Fig. 2). Antennae: ca 5.25 mm long, reaching the middle of T 6, composed of 20+19 elongate articles (Fig. 20). Ocelli: 11 on each side, dark, arranged in three broken rows; posterior ocellus slightly larger than posterosuperior ocellus and other seriated ocelli. Tömösváry’s organ as large as nearest ocellus, rounded (Figs 8, 26). Forcipular coxosternite: dental margin slightly concave, with 2+2 acute teeth and setiform porodonts, median diastema V-shaped; shoulders of coxosternite strongly sloping, as in Figs 4 and 23.

Tarsal articulation of all legs distinct (Figs 13–15). Legs 14 slightly incrassate, without visible modifications (Fig. 13). P, F, Ti and Ts1 of legs 15 incrassate, last three with modifications: F with a clearly expressed dorsal sulcus and a poorly-developed ventral one; Ti with a poorly-expressed dorsal sulcus and a well-developed ventral sulcus, the latter reaching the distoventral tubercle supporting a cluster of curved and long setae (distal setae more strongly curved, unciform, proximal ones almost straight) (Figs 12, 15); Ts1 slightly flattened, dorsally with an implicit impression (Fig. 11) bearing a few erect setae. Length of legs 15: P = 0.78, F = 0.83, Ti = 0.83, Ts1 = 0.83, Ts2 = 0.48. Legs 13–15 with DCa. Accessory claw on leg 15 large, well-developed. Plectrotaxy as in Table 1. Coxal pores: present on legs 12–15, rounded, separated from one another by distances 2–2.5 times greater than their own diameter; inner pores smaller than neighbouring ones; formula 3, 4(5), 4(5), 4. Gonopod 1-segmented, with three setae (Figs 7, 18).

**Figures 10–12.**
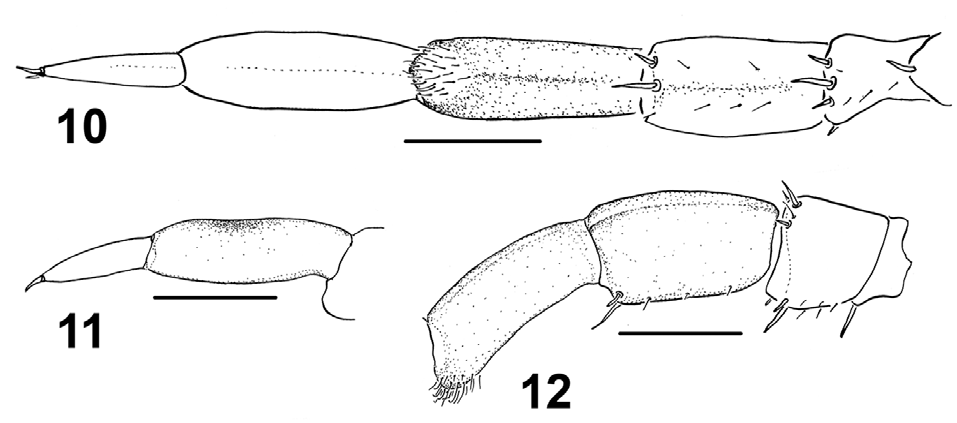
Lithobius (Chinobius) yuchernovi sp. n., male paratype. **10** leg 15, ventral view **11** tarsus 15, lateral view **12** prefemur, femur and tibia of leg 15, lateral view. Scale bars: 0.5 mm.

**Table 1.** Plectrotaxy of *L. yuchernovi* sp. n. Holotype. Brackets show the presence of an asymmetric spine in one of leg pairs.

**Leg**	**Ventral**				**Dorsal**			
	**t**	**P**	**F**	**Ti**	**C**	**P**	**F**	**Ti**
1	–	–	a m	m	–	p	a	a
2	–	–	a m	m	–	p	a p	a
3	–	–	a m	m	–	p	a p	a (p)
4	–	–	a m	m	–	p	a p	a p
5	–	–	a m	m	–	(a) p	a p	a p
6	–	–	a m	(a)m	–	a p	a p	a p
7	–	(m)	a m	a m	–	a p	a p	a p
8	–	m	a m	a m	–	a p	a p	a p
9–11	–	m p	a m p	a m	–	a p	a p	a p
12	–	m p	a m p	a m	–	a m p	a p	a p
13	–	m p	a m p	a m	a	a m p	p	p
14	m	a m p	a m	m	a	a m p	p	–
15	m	a m p	a m	–	a	a m p	–	–

Paratype ♂♂. Length 10.2–12.2 mm. All characters as in holotype, but ocelli 8–10, usually 9 (Figs 8, 26). Antennae normally with 20+20 segments, but 3 specimens with asymmetric numbers of antennal articles (19+20, 20+19 and 18+20); 6 specimens with broken antenna (20+?). Posterior and posterosuperior ocelli slightly different in size from seriate ocelli. All specimens with a well-visible distoventral tubercle, the latter carrying a group of straight or curved setae. Expression of other sulci on legs 15 variable: in some males, dorsal sulci on F and Ti poorly visible or absent at all, while in others, ventral sulci not developed. In some specimens, dorsal impression on Ts1 without erect setae (Fig. 11). Plectrotaxy as in holotype, but in some specimens Vtm can start with legs13, while VTia from legs 3 to 14. Coxal pores as in holotype, their number varying from 3 to 5. Gonopods as in holotype, with 2–3 setae (Figs 7, 18).

Paratype ♀♀. Length 12.1–16.0 mm. Antennae usually with 20+20 segments, but in two specimens 20+19 and 20+18, respectively; 3 specimens with broken antenna (20+?). Intermediate tergite broadened, breadth/length ratio 1.19 (length 0.80 mm, breadth 0.95 mm) (Fig. 9). Legs 12–15 with 4–6 coxal pores, formula 4(5),5(4),5(4),5(6). Gonopods without setae on internal face, with 2+2 short spurs separated from one another by distances not exceeding the diameter of the widest part of a spur (Figs 5, 22). First segment of gonopod without spines, second with 6–8, third with 2–3 dorsal short spines in two uneven rows (Figs 6, 24). In some specimens, the spines very short. Apical claw of gonopod with one well-defined lateral denticle located in the middle of external ridge, as well as with 2–4 blunt denticles on internal ridge. In some specimens, the denticles almost abraded. In most specimens, the lateral denticles on internal ridge of claw blunt, as in Figs 27 and 28. Other characters as in holotype.

**Figures 13–20.**
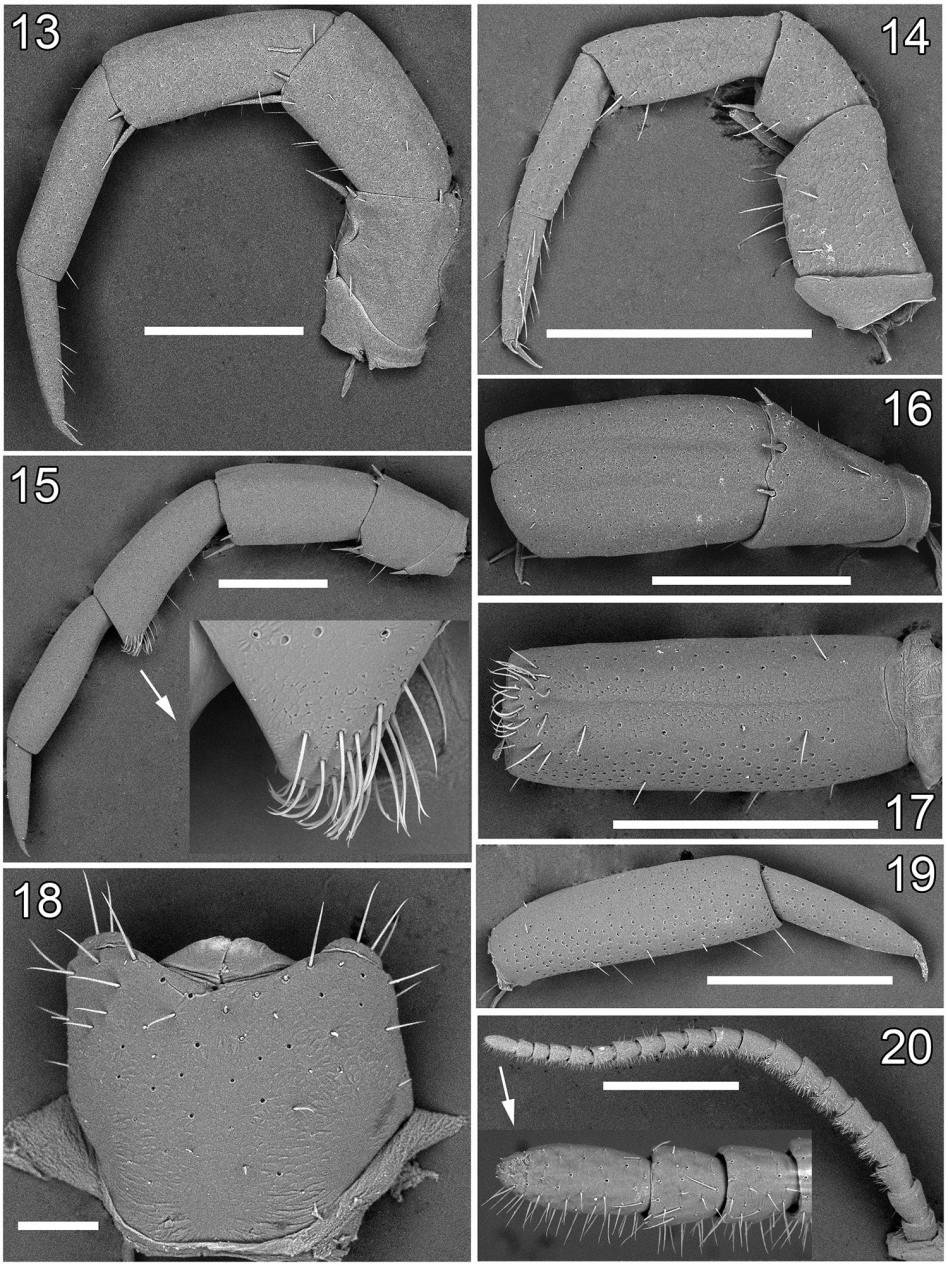
Lithobius (Chinobius) yuchernovi sp. n., male paratype. **13** leg 14, lateral view **14** leg 1, lateral view **15** leg 15, lateral view **16** prefemur and femur of leg 15, dorsal view **17** tibia 15, ventral view **18** genital sternite with gonopods, ventral view **19** tarsus 15, lateral view **20** antenna. Scale bars: 0.1 mm (**18**), 0.5 mm (**13, 14, 16, 17, 19**), 1 mm (**15, 20**).

##### Variation.

Although males from different localities in northeastern Siberia show relatively stable morphological features, females from various places demonstrate certain variability in the structure of the gonopods. Thus, in females from Chukotka, the lateral denticles on the gonopodal claw are better expressed (Fig. 25) than in the type material from the Magadan Region (Figs 5, 22). The Kamchatka specimens differ from all others to the greatest extent: both sexes are with faint, but still visible tarsal articulations on the first eleven pairs of legs (in one large male, the tarsal articulations were truly distinct); males with the distoventral tubercle on Ti 15 that supports fewer setae and less strongly expressed dorsal and ventral sulci. In females, the lateral denticle on the external ridge of the gonopodal claw is very weakly expressed, up to absent in some females. In addition, the internal ridge of the claw with one well-expressed and one almost abraded denticle, the latter looks like an uneven edge at the claw base (Fig. 28).

**Figures 21–28.**
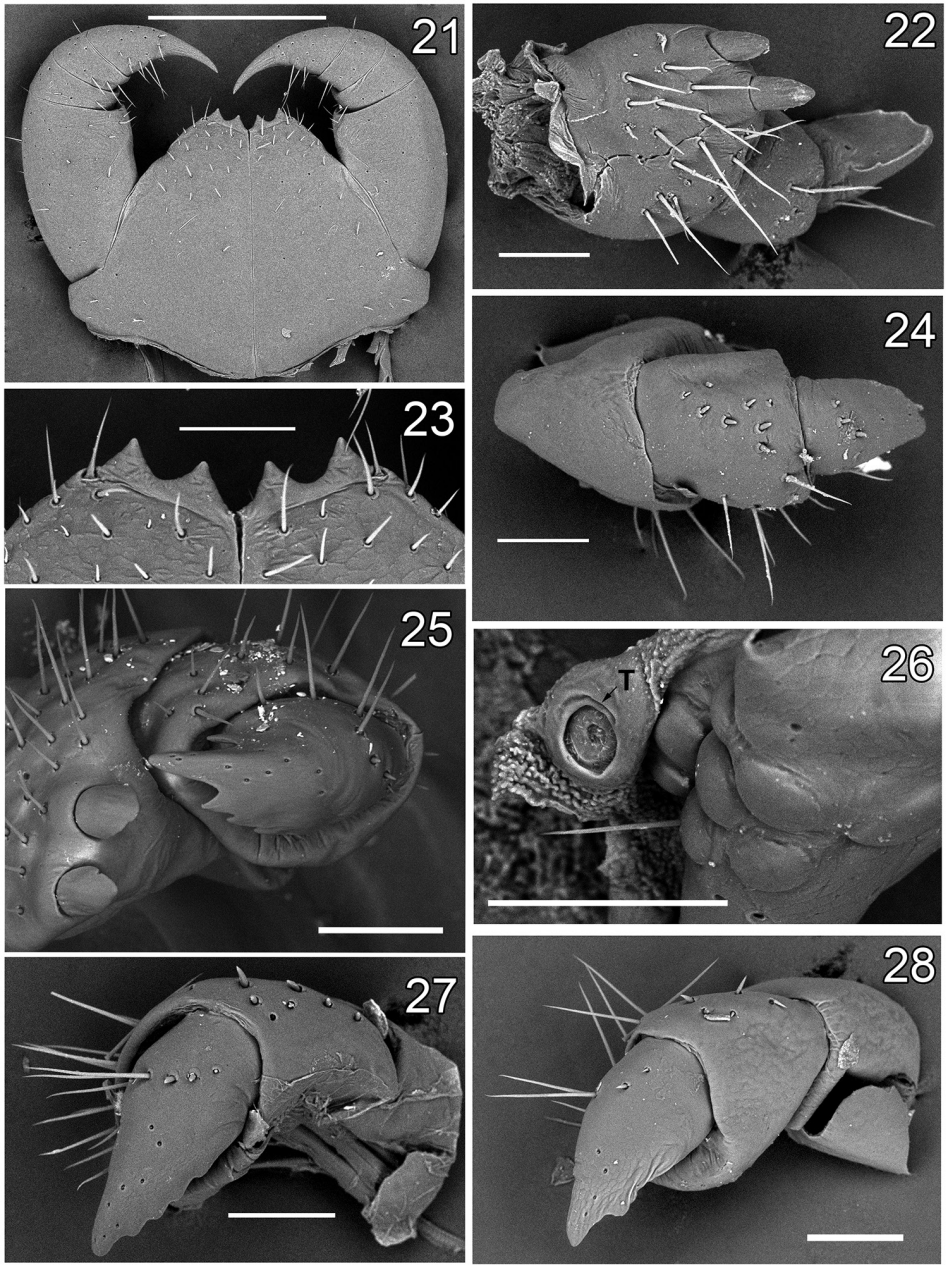
Lithobius (Chinobius) yuchernovi sp. n., female paratype (**21–24, 26**) and non-type females (25, 27–28). **21, 23** forcipule and dental margin of forcipular coxosternite, ventral view **22** gonopod, ventral view **24** gonopod, dorsal view **25** gonopod, ventrolateral view (Chukotka) **26** ocelli and Tömösváry’s organ, lateral view **27** gonopod, dorsolateral view (Magadan Region) **28** same (Kamchatka Peninsula). Scale bars: 0.5 mm (**21**), 0.1 mm (**22–28**).

##### Habitats.

Almost all specimens were collected in montane environments (800 to 1470 m), with only a few obtained from a plain area at ca 60 m near the town of Anadyr. In the mountains, the new species dwells in mountain *Betula* forest, alpine meadow, montane tundra with *Dryas* dwarf bush and *Cassiope tetragona* nival habitats.

**Figure 29.**
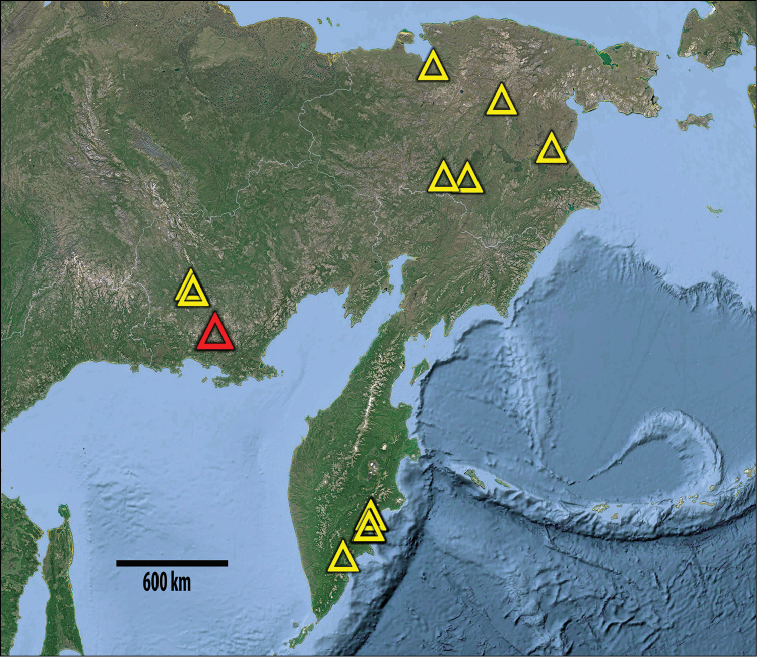
Distribution of Lithobius (Chinobius) yuchernovi sp. n. The type locality is shown in red.

## Remarks

As a nomenclatural remark, *Lithobius zachiui* nom. n., is proposed herewith as a new replacement name for Lithobius (Chinobius) orientalis (Matic, 1973). The name Lithobius (Chinobius) orientalis (Matic, 1973) must be considered as permanently invalid (ICZN 1999: Art. 57.2) since it is a junior primary homonym of *Lithobius orientalis* Sseliwanoff, 1880, even though the two were originally described in different genera (ICZN 1999: Art. 57.2). Since there are no junior synonyms, a new name, *Lithobius zachiui* nom. n., is advanced here to replace the preoccupied name *Lithobius orientalis* (Matic, 1973). The new name is dedicated to Zachiu Matic (1924–1994). Besides *Lithobius matici* Prunescu, 1966, which is a synonym of *Lithobius punctulatus* (C.L. Koch, 1847), this is the second species to honour the outstanding Romanian myriapodologist who described 193 taxa of centipedes (Bonato et al. 2016, Zapparoli and Minelli 1995).

Another nomenclatural remark, *Lithobius carli* nom. n., is proposed herewith as a new replacement name for Lithobius (Alokobius) orientalis Attems, 1953. This name must be considered as permanently invalid (ICZN 1999: Art. 57.2) since it is a junior primary homonym of *Lithobius orientalis* Sseliwanoff, 1880 too (ICZN 1999: Art. 57.2). Since there are no junior synonyms, a new name, *Lithobius carli* nom. n., is advanced here to replace the preoccupied name Lithobius (Alokobius) orientalis Attems, 1953. The new name is dedicated to Carl August Graf Attems-Petzenstein (1868–1952) to honour this prolific Austrian myriapodologist who described almost 400 taxa of centipedes (Strouhal 1960, Bonato et al. 2016).

Males of *L. yuchernovi* sp. n. differ from those of all known Palaearctic species of the genus *Lithobius* by the presence of a distoventral tubercle on Ti 15. Based on the structure of the gonopod, females are close to species of the subgenus Chinobius, from which they differ by following morphological details of the body. Thus, our new species is distinguished from L. (Ch.) orientalis Sseliwanoff, 1880 by the 20-segmented antennae (vs. 19-segmented); from L. (Ch.) zachiui nom. n. by the non-swollen gonopodal segment 2 and the presence of 6–8 dorsal short spines on it (vs. segment 2 swollen and spineless); from L. (Ch.) pectinatus Takakuwa, 1939 by the presence of a well-defined denticle on the external ridge of the claw and thick spurs (vs. without such a denticle and with very thin spurs).

Based on the structure of the female gonopod and other main characters (body length, number of antennomeres and ocelli, 2+2 coxosternal teeth, tergites without posterolateral triangular projections), *L. yuchernovi* sp. n. also resembles *L. otasanus* Takakuwa, 1941, a species described from the southern Sakhalin Island (Takakuwa 1941) and omitted by Zalesskaja (1978) in her book on the Lithobiomorpha fauna of the former Soviet Union. However, both differ clearly in the latter species showing neither DCa nor accessory claws on the last legs. Both these taxa differ also in the plectrotaxy patterns of legs 1, 13–15.

In addition, females of the new species are close to the northern Pacific species Lithobius (Ezembius) stejnegeri (Bollman, 1893) which inhabits Alaska, the Pribilof and Commander Islands, as well as some Aleutian Islands. However, *L. yuchernovi* sp. n. differs by the presence of 2 or more lateral denticles on the internal ridge of the gonopodal claw (vs. a tripartite or simple gonopodal claw in young and adult females, respectively (Zalesskaja 1978)).

Another patronym in lithobiid taxonomy that honours Academician Y.I. Chernov is worth mention. This is Lithobius (Monotarsobius) chernovi (Zalesskaja, 1976), described from the Taymyr Peninsula in the Far North of Russia. The valid name for L. (M.) chernovi is Lithobius (Monotarsobius) alticus (Loksa, 1965) (Zalesskaja 1978: 174). Although both species, viz. L. (M.) alticus and L. (Chinobius) yuchernovi sp. n., belong to different subgenera, they share an important taxonomic character, i.e. the presence of a distal tubercle supporting a cluster of long and curved setae on Ti 15. Nevertheless, they differ significantly in the position of this tubercle: distoventral in *L. yuchernovi* sp. n., vs. distodorsal in *L. alticus*.

## Discussion

Originally, Verhoeff (1934) proposed *Chinobius* as a subgenus of *Lithobius* to receive two new species from central China: *L. svenhedini* Verhoeff, 1934 and *L. hummelii* Verhoeff, 1934, the former based on a male, the latter based on a damaged female. Since neither have been chosen to serve as the type species, *Chinobius* had languished as an invalid name until Matic (1973) typified it through the selection of *L. svenhedini* as the generotype. He also elevated *Chinobius* to full generic status.

The taxonomic problems existing within *Chinobius* were discussed by Eason (1996), who again downgraded *Chinobius* to the status of a subgenus of *Lithobius*, albeit improperly ascribing the authorship of *Chinobius* to Verhoeff (Jeekel 2005: 14). At present, the main feature characterizing *Chinobius* lies in the development of two or more denticles on the internal ridge of the female gonopodal claw. However, as noted above, the females of L. (Ch.) yuchernovi sp. n. show significant variations in this trait, both intra- and interpopulational. Specimens with well-defined 2–3 lateral denticles at the internal margin of the gonopod, as well as with almost abraded or completely absent denticles are observed within and between various populations. This severely undermines the acceptance of *Chinobius* as a generic-level taxon.

On the other hand, females of L. (Ch.) yuchernovi sp. n. from the Kamchatka Peninsula are extremely similar to those of L. (E.) stejnegeri Bollman, 1893. When redescribing L. (E.) stejnegeri from Kamchatka and the Iturup Island, Kuriles, Eason (1996) recorded several specimens of both sexes which had been collected from some localities in Kamchatka and support what we identify as L. (Ch.) yuchernovi sp. n. Although the males of these two species are clearly distinguished, morphological differences between the females are quite subtle: (1) 8–9 ocelli in the new species vs. 9–18 in *L. stejnegeri*, i.e. 12–18 ocelli after Bollman (1893), but 9–15 ocelli following Eason (1996); (2) gonopodal segment 1 dorsally with a single, short, setiform spine in *L. yuchernovi* sp. n. vs. devoid of a spine in *L. stejnegeri*; (3) gonopodal segment 2 dorsally with 5–6 short and setiform spines in *L. yuchernovi* sp. n., vs. only 3 short and setiform spines in *L. stejnegeri*.

As a result, Kamchatka appears to harbour both these species which are best distinguished based on male specimens, whereas females are difficult to separate. Whether these species occur not only sympatrically, as is the case concerning the Kamchatka Peninsula, but also syntopically remains open to question.

Males of the new species are characterised by a feature unique among the Palaearctic Lithobiinae Verhoeff, 1907, i.e. a distoventral rounded tubercle supporting a group of setae on Ti 15. In addition, they show more or less strongly expressed dorsal and ventral sulci on ultimate legs. Similar distoventral modifications of male Ti 15 are observed in the North American genus *Nothembius* Chamberlin, 1916. *L. yuchernovi* sp. n. seems to be particularly close to *N. aberrans* Chamberlin, 1916 (see Chamberlin 1916, pl. 9, fig. 5), based on this feature, but differs by the number of antennomeres: 20 in *L. yuchernovi* sp. n., vs. usually 22 in *N. aberrans*; 2+2 teeth at the dental margin of the forcipular coxosternite in *L. yuchernovi* sp. n., vs. 3+3 in *N. aberrans*; the presence of sulci on the dorsal and ventral sides of Ti 15 in *L. yuchernovi* sp. n., vs. their absence in *N. aberrans*; and the claw of the female gonopod equipped with more than two lateral denticles in *L. yuchernovi* sp. n. (Figs 5, 22, 25, 27), vs. simple in *N. aberrans* (cf. Chamberlin 1916).

## Supplementary Material

XML Treatment for Lithobius (Chinobius) yuchernovi
